# Gene expression profiling of brains from bovine spongiform encephalopathy (BSE)-infected cynomolgus macaques

**DOI:** 10.1186/1471-2164-15-434

**Published:** 2014-06-05

**Authors:** Maura Barbisin, Silvia Vanni, Ann-Christin Schmädicke, Judith Montag, Dirk Motzkus, Lennart Opitz, Gabriela Salinas-Riester, Giuseppe Legname

**Affiliations:** Department of Neuroscience, Scuola Internazionale Superiore di Studi Avanzati (SISSA), Via Bonomea 265 34136 Trieste, Italy; Unit of Infection Models, German Primate Center, Kellnerweg 4 37077 Göttingen, Germany; Microarray Core Facility, University Medical Center Göttingen, Justus-von-Liebig-Weg 11 37077 Göttingen, Germany; Molecular and Cell Physiology, Hannover Medical School, Carl-Neuberg Str. 1 D-30625 Hannover, Germany

**Keywords:** Prion diseases, BSE, Non-human primates, Neurodegeneration, Transcriptome, Microarray, RT-qPCR, Biomarker, Serpina3, Hemoglobin

## Abstract

**Background:**

Prion diseases are fatal neurodegenerative disorders whose pathogenesis mechanisms are not fully understood. In this context, the analysis of gene expression alterations occurring in prion-infected animals represents a powerful tool that may contribute to unravel the molecular basis of prion diseases and therefore discover novel potential targets for diagnosis and therapeutics. Here we present the first large-scale transcriptional profiling of brains from BSE-infected cynomolgus macaques, which are an excellent model for human prion disorders.

**Results:**

The study was conducted using the GeneChip® Rhesus Macaque Genome Array and revealed 300 transcripts with expression changes greater than twofold. Among these, the bioinformatics analysis identified 86 genes with known functions, most of which are involved in cellular development, cell death and survival, lipid homeostasis, and acute phase response signaling. RT-qPCR was performed on selected gene transcripts in order to validate the differential expression in infected animals versus controls. The results obtained with the microarray technology were confirmed and a gene signature was identified. In brief, *HBB* and *HBA2* were down-regulated in infected macaques, whereas *TTR*, *APOC1* and *SERPINA3* were up-regulated.

**Conclusions:**

Some genes involved in oxygen or lipid transport and in innate immunity were found to be dysregulated in prion infected macaques. These genes are known to be involved in other neurodegenerative disorders such as Alzheimer’s and Parkinson’s diseases. Our results may facilitate the identification of potential disease biomarkers for many neurodegenerative diseases.

**Electronic supplementary material:**

The online version of this article (doi:10.1186/1471-2164-15-434) contains supplementary material, which is available to authorized users.

## Background

Prion diseases, or transmissible spongiform encephalopathies (TSEs), are incurable and fatal neurodegenerative disorders that affect both humans and animals; their origin may be sporadic, acquired or genetic [1, 2]. TSEs include Creutzfeldt-Jakob Disease (CJD), Gerstmann-Sträussler-Scheinker syndrome (GSS), kuru and fatal familial insomnia (FFI) in humans [2], bovine spongiform encephalopathy (BSE) in cattle [3], scrapie in sheep and goats [4], chronic wasting disease (CWD) in cervids [5], transmissible mink encephalopathy, and feline spongiform encephalopathy (FSE) [6].

A major event that leads to the development of prion diseases is the conversion of the cellular form of the prion protein (PrP^C^) into an abnormally folded, β-sheet enriched and protease resistant isoform (PrP^Sc^). PrP^Sc^ is prone to accumulate and aggregate in the brain of affected individuals [1, 2, 4] leading to neuronal loss, spongiosis and astrogliosis, which are hallmarks of neurodegeneration. The underlying conversion mechanism of PrP^C^ into PrP^Sc^ is poorly understood and it is further complicated by the existence of several different strains characterized by distinct tertiary and quaternary structures as well as different clinical patterns [7, 8]. Several hypotheses exist about the contribution of unknown molecules other than PrP to prion propagation [9–11]. To address this issue, several animal studies have investigated the host response to prion infection of different origin and strain. The differential transcription profile after prion infection has been extensively explored (reviewed in [6, 12]); however, most of the studies involved animal models such as mice [13–18], sheep [4, 19–22] and cattle [23–28], all not closely related to humans. Some expression analyses have been conducted in non-human primates focusing mainly on the susceptibility to the infection and the variety of clinical symptoms [29–33], but none has investigated large-scale transcriptome changes due to prion infection. All these investigations suggest that besides the PrP-encoding gene (*PRNP* in humans), other genes are key players and contribute to the genetic susceptibility to acquired TSEs [6, 34]. The main genes identified so far are related to oxidative stress, mitochondrial apoptotic pathways, endosome/lysosome function, immunity, synapse function, metal ion binding, activated cholesterol biosynthesis, immune and inflammatory response, protease inhibitors, calcium binding proteins, regulation of the actin cytoskeleton, ion transport, cell adhesion, and transcription processes [6]. Dysregulation of these genes seems to cause increased oxidative stress that in turn determines oxidation of proteins, lipids and DNA as well as mitochondrial dysfunction and ER stress [6]. Apart from TSEs, transcriptional changes of these genes are common to other neurodegenerative pathologies [12] and, together with functional proteomics data, may help to identify novel selective biomarkers of prion diseases and neurodegeneration in general.

To accomplish that, we performed a large-scale transcriptional profiling in BSE-infected cynomolgus macaques (*Macaca fascicularis*). They are known to be an excellent model for studying human acquired prion diseases [32, 33, 35–37], as shown by BSE transmission via the intracranial and oral routes, which lead to a disease pattern comparable to that of human maladies in terms of preclinical incubation time, clinical symptoms and pathophysiology [35]. The objective of this study was to identify genes that are differentially expressed in brain tissue of intracranially infected monkeys compared to non-infected ones using an unbiased genomic approach such as expression microarrays with subsequent data validation by RT-qPCR. Our study aims at revealing biological processes that are relevant to the pathogenesis of human prion diseases using a systematic approach that connects the identified DEGs into potential networks of interacting pathways. This may allow us to discover novel selective markers as potential targets for diagnostic and therapeutic strategies.

## Results

### PrP^Sc^ content in brain tissue

**Figure 1:**
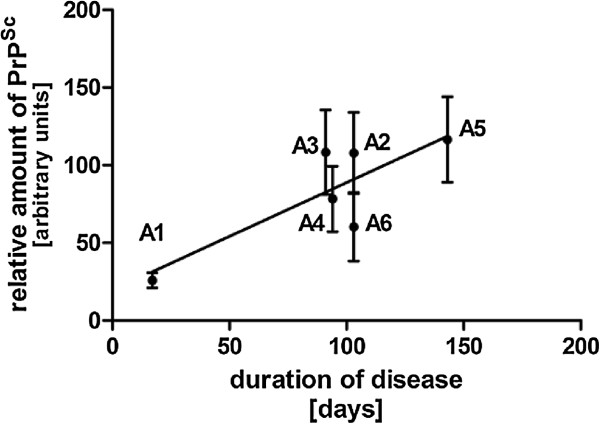
**Correlation between PrP** ^**Sc**^ **content and duration of clinical phase.** Western Blot analysis from PK-treated homogenates of brain samples derived from BSE-infected macaques was performed. The monoglycosylated bands of PrP^Sc^ were analyzed densitometrically. Relative amounts of PrP^Sc^ from brain homogenates were averaged and correlated to the disease duration.

The relative amount of PrP^Sc^ in brain homogenate of 6 BSE-infected macaques was examined by Western Blot. Densitometric analysis of the monoglycosylated band revealed that the relative amount of PrP^Sc^ strongly differed between the individual macaques. We wondered whether this discrepancy might be due to the preclinical incubation time or rather correspond to the gradual accumulation of PrP^Sc^ during the clinical phase of disease as reported for sCJD [38, 39]. As anticipated, we found a significant correlation between PrP^Sc^ content and the duration of the symptomatic phase (Figure 1). The correlation analysis includes only the 6 intracranially inoculated macaques. Since these animals were housed in one social group, environmental factors, which may influence the disease course and duration, are identical. Such factors can be different for the orally inoculated animal, which was therefore omitted from the analysis. The infected animals were at an advanced stage of prion disease and the details of their clinical course have been previously described [33]. Briefly, animal A1 showed the shortest duration of disease (17 days) and a short pre-clinical incubation time (931 days) together with the lowest PrP^Sc^ content, while animal A5 showed the longest survival period (143 days), compared to an average clinical phase of about 90 days, together with the highest PrP^Sc^ content and the second longest pre-clinical phase (1340 days).

### Microarray analysis of brain gene expression in cynomolgus macaques

To investigate differential mRNA expression in BSE-infected macaques we used brain samples from 6 animals that were intracranially challenged [33]. One macaque that was orally infected with 50 mg BSE-homogenate was also included in our study. For comparison purposes, we used 5 brain samples derived from non-infected age- and sex-matched control macaques.

RNA was isolated from the *gyrus frontalis superior* of all animals and checked for quality by nano-scale electrophoresis, which resulted in an overall RNA Integrity Number (RIN) of about 6. This value is indicative of at least partially degraded RNA within the sample; one possible reason for the reduced RNA integrity may be the procedure utilized to remove the *gyrus frontalis superior* region from the frozen tissue slide. The biopsy stamp was plugged into a cordless screwdriver that was used to drill a borehole in the frozen tissue block of +/− 1 cm height. This method was chosen to ensure that the material did not thaw; however, the local heat induced by the rotating biopsy stamp may have led to substantial degradation of the RNA. Nonetheless, human brain material exhibiting a comparable RIN value was successfully used for similar studies [40]. All samples were analyzed using the GeneChip® Rhesus Macaque Genome Array (Affymetrix®) that contains 52,024 rhesus probe sets to enable gene expression studies of *Macaca mulatta* transcriptome interrogating more than 47,000 transcripts. The genomes of *M. mulatta* and *M. fascicularis* exhibit a small genetic divergence of approximately 0.4% [41, 42] that presumably allows for the detection of homologue transcripts with high specificity.

**Figure 2:**
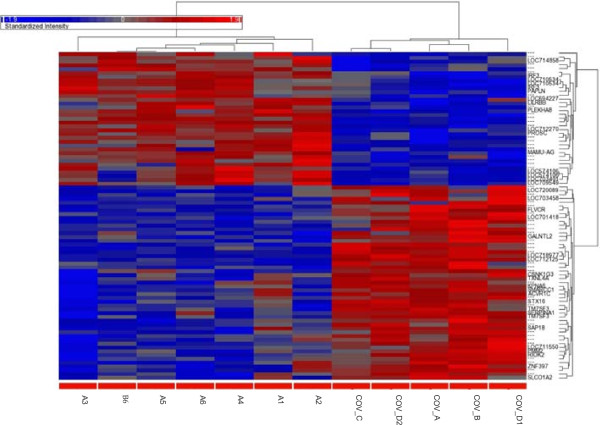
**Condition trees of the clustering analysis.** The cluster analysis was performed using a hierarchical approach with the average linkage-method (R and Partek® Software, Partek® Inc.): 86 probe sets showed a differential expression with FC ≥ 2. The color represents the level of expression (red: up-regulation, blue: down-regulation) and the sample information is listed across the bottom. The names of the known genes are indicated. More details on all genes are reported in Additional file 1.

Raw data were quality checked and analyzed using Affymetrix® proprietary analysis tools, a hierarchical clustering was performed and a heat map was generated. Then the signals were aligned to the annotation library and a spreadsheet containing gene symbols, p-values and expression fold changes was created. Microarray data were submitted to Gene Expression Omnibus (GEO). The bioinformatics analysis identified 300 probe sets that were up- or down-regulated about twofold (≥ |1.95|). Because among them no candidate appeared using FDR 0.05, we chose as criteria an unadjusted p-value of ≤ 0.005 together with a fold change ≥ |2.0|. Additional file 1 lists the resulting 86 probe sets that were then used to generate the heat map shown in Figure 2.

### Functional classification of differentially expressed genes (DEGs)

We used the Ingenuity Pathways Analysis (IPA®, see section: Availability of supporting data) to annotate genes according to their functional relationships and to determine potential regulatory networks and pathways. Among the 300 differentially expressed (about twofold, (≥ |1.95|) probe sets identified, 105 were associated to mapped IDs; 53 of the latter were identified as network eligible genes, while 86 were identified as function eligible genes. It should be emphasized that the designation of functional class in the present study is neither definitive nor exclusive, as annotation of gene function is incomplete, and multifunctional gene products can be involved in several cellular pathways. First, we identified key biological functions and/or diseases that contain a disproportionately high number of genes from the DEG list compared to the total gene population from the microarray. The analysis was started by identifying the top categories (p < 0.01) of DEGs within three main classes. In the “Diseases and Disorders” class the categories were cancer and developmental disorder, while within the “Molecular and Cellular functions” class most genes were involved in cellular development and cell death/survival. The main categories for the “Physiological System Development and Function” class were tissue morphology as well as nervous system development and function. As a second step, genes were clustered in relation to the main pathways they belong to: the top two canonical pathways in our DEG list were LXR/RXR activation, which is associated with lipid metabolism and transport, and acute phase response signaling.

### Identification of biologically relevant networks

**Figure 3:**
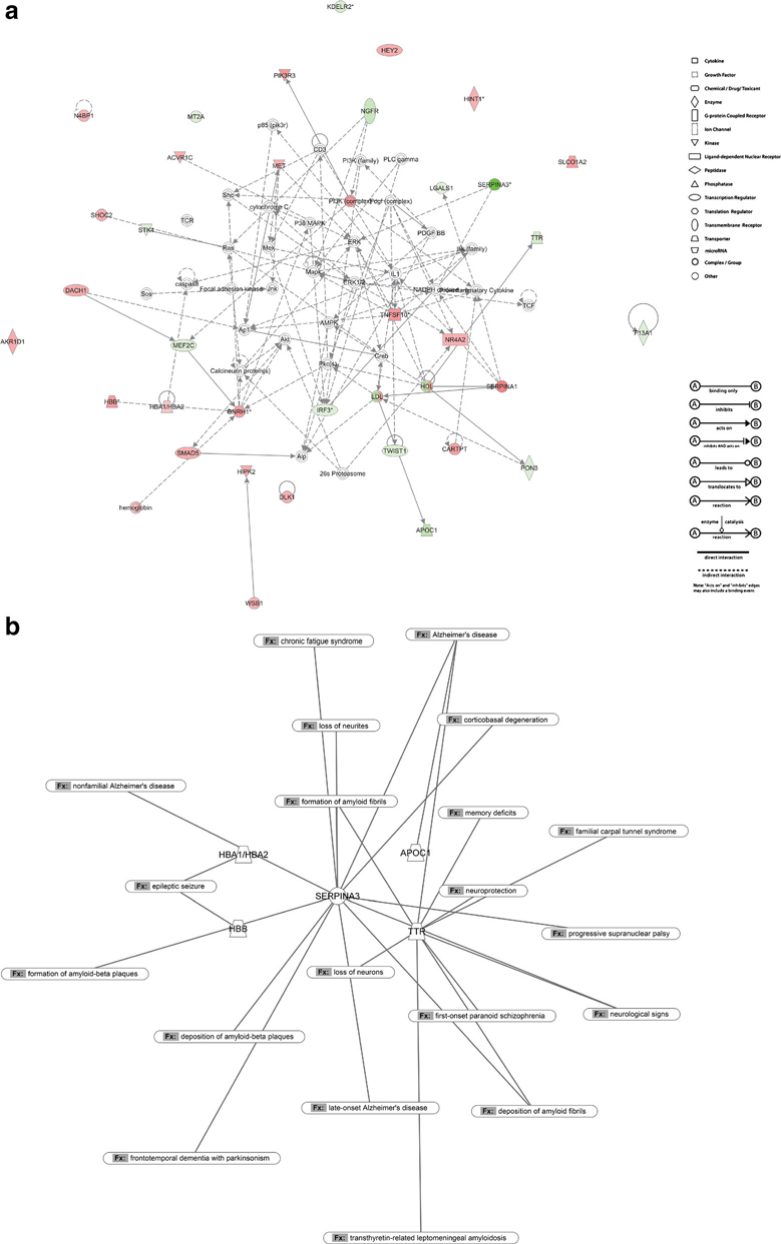
**Identification of biologically relevant networks. (a)** Top ranking network generated by mapping the focus genes that were differentially expressed in infected animals. Pathway analysis based on the Ingenuity Pathway Knowledge Base (IPKB) is shown. Color shading corresponds to the type of dysregulation: red for up-regulated and green for down-regulated genes according to the microarray fold change calculation method. White open nodes are not from the list of 300 DEGs, but are transcription factors that are associated with the regulation of some of these genes identified by IPKB. The shape of the node indicates the major function of the protein. A line denotes binding of the products of the two genes, while a line with an arrow denotes 'acts on'. A dotted line denotes an indirect interaction. **(b)** Schematic representation of nervous system-related functions for selected DEGs. The most regulated/interesting DEGs were selected and associated to known nervous system-related functions according to the Ingenuity Pathway Knowledge Base (IPKB) software.

To further investigate the global expression response to BSE infection and to define interactions among the identified specific pathways containing the regulated genes, potential networks of interacting DEGs were identified using IPA®. All potential networks with score > 9 (a score ≥ 3 was considered significant, p < 0.001) are listed in Table 1 with information on network genes, score, focus molecules and top functions associated with the focus genes in each network. The highest ranked network identified by IPA® was associated with tissue morphology (specifically the determination of cell quantity), developmental disorder and biological processes controlling cell death and survival (Figure 3a). This network contained genes that are known to be involved in several neurological diseases and nervous system functions, as shown in Figure 3b.

**Table 1:** **List of 3 Ingenuity networks generated by mapping the focus genes that were differentially expressed between non-infected and BSE-infected samples**

ID	Molecules in network	Score	Focus molecules	Top functions
1	ACVR1C, AKR1D1, Alp, AMPK, Ap1, APOC1, Calcineurin protein(s), CARTPT, caspase, CD3, CHI3L1, Creb, cytochrome C, DACH1, DLK1, ERK, ERK1/2, F13A1, Focal adhesion kinase, GNRH1, HBA1/HBA2, HBB, HDL, hemoglobin, HEY2, HINT1, HIPK2, Ikk (family), IL1, IRF3, Jnk, KDELR2, LDL, LGALS1, Mapk, MEF2C, Mek, MET, MT2A, N4BP1, NADPH oxidase, NGFR, NR4A2, OTX2, P38 MAPK, p85 (pik3r), Pdgf (complex), PDGF BB, PI3K (complex), PI3K (family), PIK3R3, Pkc(s), PLC gamma, PON3, Pro-inflammatory Cytokine, Ras, SERPINA1, SERPINA3, Shc, SHOC2, SLCO1A2, Sos, STK4, TCF, TCR, TNFSF10, TTR, TWIST1, Vegf, WSB1	71	35	Tissue Morphology, Cell Death and Survival, Developmental Disorder
2	ABR, ACTL6B, ARMC6, ASB6, C10orf137, C6orf211, CAMKV, CHMP2A, CLIC4, CLPP, CSNK1G3, CTBP2, DCLRE1A, DDX19B, DGKE, ECT2, FHL3, FLVCR1, GALNTL5, GLOD4, HEATR6, HSP90AA1, HSPA12A, ITFG1, KLF3, KPNA6, MCTS1, MEIG1, METTL7B, MRPL44, MXD3, MYBPC1, NCLN, NIPBL, NOL4, OSBPL10, PCBP3, PLEKHA8, PMM2, POLR2J, PPAP2C, PRCP, PROSC, RAI2, SAP18, SCAND1, SEPT6, SGTB, SMARCC1, SMC3, SPATA22, SPSB3, SRPK3, SSU72, STAG1, TATDN1, TESPA1, TM7SF3, TNK1, TNNI3K, TP53BP1, TRAPPC2L, TRIP12, TUFM, TXNL4A, UBC, ZNF131, ZNF235, ZNF397, ZNF420	54	28	Developmental Disorder, Hereditary Disorder, Hematological Disease
3	26 s Proteasome, ADCY, AKR1C1/AKR1C2, Akt, APP, ARL4C, Arntl-Clock, AVP, AVPR1B, CACNA1B, CAMKV, CARTPT, CBLN2, CEACAM6, CLDN10, CLOCK, COX4I2, CTF1, DNAJC12, endocannabinoid, estrogen receptor, FAM46A, FSH, GABRE, GNA15, GPR158, GPX1, GPX2, GSK3A, Histone h3, HMGCR, HNF4A, HSPA12A, Insulin, JPH3, KCNC3, KCNS1, LINGO1, LPAR1, LXN, MGAT2, miR-125b-5p (and other miRNAs w/seed CCCUGAG), Mmp, MST1, NFkB (complex), Npff, OPN1LW, PDX1, PIK3R5, Pka, PKM, PLC, Proinsulin, RAB39A, RAI2, RIOK2, RUFY3, SERPINA3, SMAD5, SMC4, SOX7, SYT17, TCF19, Tnfrsf22/Tnfrsf23, TOR2A, tretinoin, trypsin, TXNL4B, ZBTB44, ZFHX3	36	21	Cellular Development, Neurological Disease, Skeletal and Muscular System Development and Function

Names in lowercase are genes/molecules that are not from the DEG list but are associated with some of them within pathways identified by Ingenuity Pathway Knowledge Base (IPKB).

### Validation of differentially expressed genes by RT-qPCR

To further confirm the array results using an independent and more sensitive technique, we decided to perform RT-qPCR for a subset of differentially expressed genes. This subset (Additional file 2) was selected in subsequent steps: first, among the 86 probe sets identified during the microarray analysis (Additional file 1) we selected the top 36 with fold change ≥ |2.5| and p ≤ 0.005. Then, after realizing that many were not annotated or did not have a known function, we extended the selection to additional 29 probe sets having fold change ≥ |2.5| but 0.005 ≤ p ≤ 0.05; for the same reasons stated above, we extended the list of candidates one more time using as criteria fold change ≥ |2.5| and p > 0.05 (24 candidates). At this point, having still some cDNA available and only 13 feasible candidate transcripts, we added seven probe sets, corresponding to 5 additional transcripts, selected among the ones with a slightly lower fold change (FC ≥ 2 for at least 1 probe) but possessing an interesting function as revealed by the IPA® analysis or according to the literature. Lastly, *HBA2* was added to the list because of its tight relationship with one of the previously selected genes of the hemoglobin complex (*HBB*), as revealed in the top ranking network from the IPA® analysis (Figure 3a). In summary, we designed RT-qPCR assays for 19 genes (Table 2) and most of them were already known to be involved in neurodegenerative disorders or nervous system regulation, even though very few had been implicated in prion diseases. Among these, we were able to successfully analyze only 11 (reported in Table 3 together with 2 housekeeping genes, *ACTB* and *GAPDH*), since the RT-qPCR assays for the remaining 8 genes either showed too low expression (C_T_ > 35) or amplification of trace amounts of residual gDNA. Furthermore, because several gene names have changed since the first annotation was done, updated names from the latest Affymetrix® annotated library are provided in Additional file 2, together with the old ones.

**Table 2:** **Candidate genes for validation**

Gene	Accession number	Known relation with PrP/nervous system	References
**AKR1C1**	NM_001195574.1	Putative role in myelin formation	[43]
**HBB**	NM_001164428.1	Putative role in intraneuronal oxygen homeostasis, reduced in Alzheimer's and Parkinson's disease	[44]
**NCAM1**	XM_001083697.2	PrP/N-CAM complexes found in prion infected N2a cells	[45]
**NR4A2**	NM_001266910.2	Mutations related to dopaminergic dysfunction, including Parkinson schizophrenia and depression	[46, 47]
**USP16**	NM_001260999.2	Depletion of USP16 prevented ATMi from restoring transcription after DSB induction	[48]
CALB1	XM_001085269.2	Plays a protective role in neurodegenerative disorders (depleted in HD)	[49]
DACH1	XM_001082371.2	Required for normal brain development	[50]
LXN	NM_001266988.1	Marker for the regional specification of the neocortex	[51]
PIK3R3	NM_001266826.1	Linked to β-amyloid plaque formation in AD brain	[52]
**SAP18**	NM_001261034.1	Possibly related to AD	[53]
**SERPINA3**	NM_001195350.1	Increased in schizophrenia, SNPs affecting onset and duration of AD	[54, 55]
TNFSF10	NM_001266034.1	Implicated in pathogenesis of MS (causing demyelination)	[56]
**HBA2**	NM_001044724.1	Putative role in intraneuronal oxygen homeostasis, reduced in Alzheimer's and Parkinson's diseases	[44]
GNRH1	NM_001195436.1	Key regulator of the reproductive neuroendocrine system in vertebrates	[57]
**IRF3**	NM_001135797	Putative protective role against prion infection	[58]
**APOC1** *	AK240617.1	Binds to ApoE, risk factor for Alzheimer's disease	[59]
TM7SF3	XM_001099269.2	-	-
MYBPC1	XM_001091952.1	-	-
**TTR**	NM_001261679	Amyloid neuropathies, interaction with Aβ	[60]

List of 19 identified genes selected on the basis of fold change value and known relevance for neurodegeneration. Because of very low signal (*LXN, PIK3R3, TNFSF10, GNRH1*) or lack of reliable sequence data (*CALB1, DACH1, TM7SF3, MYBPC1*), only 11 genes (in bold) were successfully analyzed. *Macaca fascicularis transcript.

**Table 3:** **Genes analyzed by RT-qPCR**

Gene	Chromosome	Primer sequence		Amplicon length (bp)	Accession number
ACTB	3	F:	GTTGCGTTACACCCTTTCTTG	146	NM_001033084.1
		R:	CTGTCACCTTCACCGTTCC		
GAPDH	11	F:	CCTGCACCACCAACTGCTTA	74	NM_001195426.1
		R:	CATGAGTCCTTCCACGATACCA		
AKR1C1	9	F:	CCGCCATATTGATTCTGCTCAT	132	NM_001195574.1
		R:	TGGGAATTGCACCAAAGCTT		
HBB	14	F:	GTCCTCTCCTGATGCTGTTATG	102	NM_001164428.1
		R:	TTGAGGTTGTCCAGGTGATTC		
NCAM1	14	F:	GAGCAAGAGGAAGATGACGAG	150	XM_001083697.2
		R:	GACTTTGAGGTGGATGGTCG		
NR4A2	12	F:	CCAGTGGAGGGTAAACTCATC	145	NM_001266910.2
		R:	AGGAGAAGGCAGAAATGTCG		
USP16	3	F:	GCAGAACTTGTCACAAACACC	146	NM_001260999.2
		R:	CTAAAGTAAGAGGGCCTGGAG		
SAP18	17	F:	GGAAATGTACCGTCCAGCGA	109	NM_001261034.1
		R:	TGCCCTTCTTTCTAGCTTCTGG		
SERPINA3	7	F:	GCTGGGCATTGAGGAAGTCT	123	NM_001195350.1
		R:	GTGCCCTCCTCAGACACATC		
HBA2	20	F:	CGACAAGAGCAACGTCAAGG	126	NM_001044724.1
		R:	TCGAAGTGGGGGAAGTAGGT		
IRF3	19	F:	TGGGTTGTGTTTAGCAGAGG	90	NM_001135797
		R:	GAAAAGTCCCCAACTCCTGAG		
APOC1*	19	F:	TTCTGTCGATGGTCTTGGAAG	138	AK240617.1
		R:	CACTCTGTTTGATGCGGTTG		
TTR	18	F:	TCACTTGGCATCTCCCCATTC	114	NM_001261679
		R:	GGTGGAATAGGAGTAGGGGCT		

Primers (F: forward and R: reverse) used for gene amplification, amplicon length and GenBank® accession numbers of the macaque cDNA sequences used for primer design. All primers were designed according to the genome sequence of *Macaca mulatta.*

*Apolipoprotein C-I (*APOC1*) primers were designed according to the genome sequence of *Macaca fascicularis* because the *Macaca mulatta* mRNA sequence was not annotated (TSA *Macaca mulatta* Mamu_450725, accession number: JV045807.1). Homology between the two sequences was 99%.

In order to achieve optimal RT-qPCR conditions we performed titration of template and primers as well as optimization of cycling conditions using human cDNA from SH-SY5Y neuroblastoma cells (macaque cDNA was scarce). To assess the specificity of the chosen oligonucleotides prior to performing the quantitative assays, some reactions were carried out using macaque cDNA obtained from control animals to verify the correct amplicon length. Two housekeeping genes, *GAPDH* and *ACTB*[61], were used as reference genes to normalize RT-qPCR data. Both genes were monitored across samples derived from infected and control macaques in order to evaluate their expression stability, yielding very similar results (Additional file 3).

At this point we performed the quantitative analysis and in general we observed large intra-assay variability for most genes across different samples, both for infected (Additional file 4) and for control animals (Additional file 5). Interestingly, we found a completely different expression pattern for B6, the only orally-infected sample, compared to the intracranially infected animals, except for a couple of genes (*AKR1C1*, *NCAM1*), suggesting that the route of infection might play a role in determining the gene expression changes (Additional file 6). Therefore we decided to rerun the microarray clustering analysis excluding this animal in order to verify its influence on the final results. As shown in Additional file 7, the comparison of the clustering analysis with (panel A) and without (panel B) the orally challenged animal B6 does not show marked differences.

**Figure 4:**
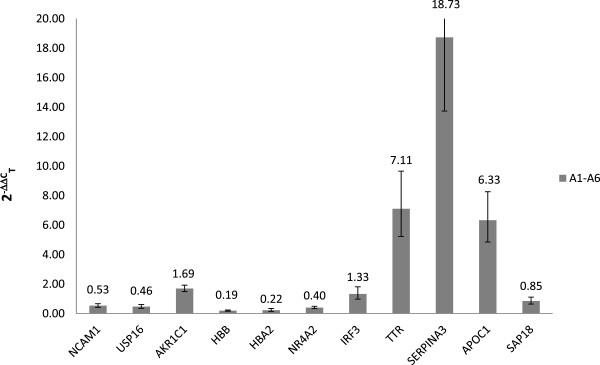
**SYBR® Green-based RT-qPCR validation of microarray results.** Relative expression levels of 11 genes normalized against *GAPDH* in BSE-infected cynomolgus macaques.

Using SYBR® Green-based RT-qPCR we confirmed the statistically significant up-regulation of *TTR* (FC = 7.11), *SERPINA3* (FC = 18.73) and *APOC1* (FC = 6.33) as well as the down-regulation of *HBB* (FC = 0.19) and *HBA2* (FC = 0.22), normalizing the data against *GAPDH* (Figure 4). Similar results were obtained against *ACTB* (Additional file 8). For all the other genes the RT-qPCR results confirmed the regulation trend of the microarrays, but without statistical significance (p-value > 0.05).

**Figure 5:**
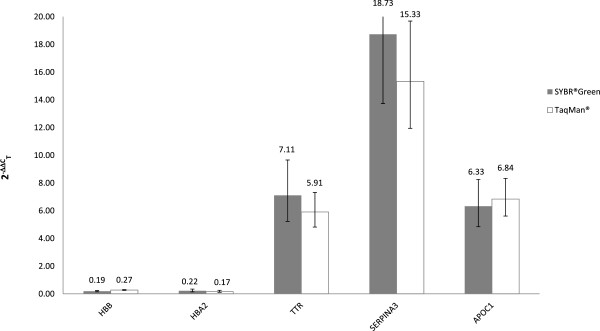
**Comparison between SYBR® Green-based and TaqMan® probe-based results.** TaqMan® (white) versus SYBR® Green-based (grey) expression levels for each transcript. Both detection systems yielded similar results. Data are normalized against *GAPDH*. Similar results were obtained with normalization against *ACTB* (data not shown).

In order to confirm the SYBR® Green -based results we performed an additional RT-qPCR analysis using FAM-labeled TaqMan® probes, providing more sensitive and specific detection signals for those genes that showed a significant fold change. Using this approach we confirmed the regulation of *SERPINA3*, *APOC1*, *HBB* and *HBA2*, but not of *TTR*, which showed comparable trends in FC but lost statistical significance (Figure 5). This may be due to higher variability among triplicates, caused by C_T_ values higher than 35 obtained with the TaqMan® probe chemistry compared to SYBR® Green detection system (Additional file 9).

In general, we were able to confirm the results of the array platform obtaining consistent fold change values for all genes analyzed, even though we validated with statistical significance using the specific TaqMan® detection system only four of them: *HBB, HBA2, APOC1, SERPINA3* (see Table 4 for details on p-values and FC).

**Table 4:** **RT-qPCR confirmation of microarray results**

Gene symbol	Microarray fold change			RT-qPCR fold change			
				SYBR® Green		TaqMan®	
	Min	Max	Mean	FC	P value	FC	P value
AKR1C1	2.3	2.9	2.5	1.7	0.433	2.4	0.235
**HBB**	−2.2	−2.6	−2.4	0.2	0.020	0.3	0.021
NCAM1	−1.1	2.5	−0.3	0.5	0.160	-	-
NR4A2	1.1	−2.1	−1.6	0.4	0.248	-	-
USP16	−1.2	−5.5	−2.6	0.5	0.308	-	-
SAP18	−1.2	−2.6	−1.7	0.8	0.393	-	-
**SERPINA3**	10.0	16.0	13.0	18.7	0.0001	15.3	0.0005
**HBA2**	-	-	-	0.2	0.041	0.2	0.019
IRF3	2.0	2.1	2.0	1.3	0.123	-	-
**APOC1**	4.3	-	4.3	6.3	0.047	6.8*	0.028*
**TTR**	3.1	-	3.1	7.1	0.025	5.9	0.076

Differential expression of selected genes analyzed by microarray and RT-qPCR. For microarray analysis, the lowest (Min), the highest (Max) and the average (Mean) fold change values of all the respective probes are shown. For RT-qPCR analysis, fold change (FC) and statistical significance (p-value) for both SYBR® Green and TaqMan® results are shown. In bold are the genes validated with statistical significance. HBA2 was not present in the array chip.

*Normalization performed vs. *ACTB* only.

In addition, dealing with animals whose brain material isolation may be susceptible to blood contamination, and as several works in the last few years have shown the presence of active transcription within human red blood cells [62], we decided to analyze the samples also for expression of some erythrocyte markers, such as *ALAS2* and *RHAG*, in order to verify the reliability of the results related to the regulation of both chains of hemoglobin (*HBB* and *HBA2*). Although the array data for these genes suggested a negligible and virtually identical presence of blood in both control and infected samples, RT-qPCR analysis revealed a small blood contamination (C_T_ ≥ 34 for *ALAS2*, C_T_ ≥ 36 for *RHAG*) within two samples, one control (CovD1) and one infected sample (A4) (Additional file 10 and Additional file 11). In light of these results, we performed an additional gene expression analysis for *HBB* and *HBA2* excluding these two samples. As expected, we obtained slightly different results (FC ~ 0.3 for *HBB* and 0.2 for *HBA2* using TaqMan® probes), but a relevant down-regulation still persisted with statistical significance.

## Discussion

The precise mechanisms regulating the molecular processes that lead to neurodegeneration in TSEs remain unknown. Genomic approaches represent unbiased and powerful tools to uncover the molecular basis of these complex mechanisms and they may also contribute to discover new biomarkers for these diseases. Several studies have presented genomic analyses of brain tissues from animal models of TSE; a few of them involved the mRNA profiling of cattle BSE [23, 25–27] or ovine scrapie [4, 19–22, 63] whereas the vast majority was performed on rodent-adapted models of prion disease [13–18, 64]. In several of these prion-infected mice, genomic expression profiles revealed the induction of oxidative and endoplasmic reticulum (ER) stress, activated ER and mitochondrial apoptosis pathways as well as activated cholesterol biosynthesis in the CNS of preclinical mice [64].

We report here the first large-scale transcriptome analysis of the superior frontal gyrus of BSE-infected macaques. This region was selected based on its histopathological and functional relevance in the majority of neurodegenerative disorders [65] and because it corresponds to Brodmann areas 10 and 11, known to be involved in strategic processes in memory recall, various executive functions as well as in planning, reasoning, and decision making [66], all processes known to be disturbed by neurodegeneration. In general, RT-qPCR results confirmed the regulation trend seen in the microarray platform for all the 11 genes analyzed with very similar values using either *GAPDH* or *ACTB* for normalization. For five of them (*HBB*, *HBA2*, *TTR*, *SERPINA3*, *APOC1*) we obtained statistical significance with one or both qPCR detection systems utilized in this study (SYBR® Green and TaqMan® probes) and some of them were involved in the top two canonical pathways identified during the functional classification reported in the Results section: *APOC1* and *TTR* are part of the LXR/RXR activation pathway, which is associated with lipid metabolism and transport, whereas *SERPINA3* and *TTR* are involved in the acute phase response signaling pathway. All the other genes seemed to fall in the grey zone of both platforms and therefore their FC values could not be considered reliable.

When validating the array results by RT-qPCR, the first evidence obtained was a marked variability among the samples of the same group, either control or infected animals. Unlike other animal models, nonhuman primates are usually not inbred. Therefore, differences in the genomic background of the animals in our study may have contributed to the variability in the time of disease onset [33] and in gene expression within the same group. Paradoxically, for some genes that resulted strongly regulated (*APOC1*, *HBB*, *HBA2*) the variability resulted even more accentuated within the control group if compared to that of the infected group. The experimental and control animals were housed in different animal facilities and this may have generated slight differences in diet and/or housing conditions that may have contributed to the above-mentioned effect.

We also reported a peculiar dysregulation pattern of the orally infected sample (B6) for several genes, showing a completely opposite trend compared to intracranially infected animals. Although no data are available for PrP^Sc^ deposition in brain or other tissues of this animal, the significantly longer incubation period (1950 days compared to an average of 1100 days for the other animals) could suggest a correlation between the mRNA expression profile and the route of infection [67]. Nonetheless, this different pattern may be due to the age difference at the time of euthanasia: 7.1 +/− 0.7 years for the intracranially infected macaques versus 9.9 years for the orally infected animal.

Concerning hemoglobin (Hb), a few years ago its expression was unexpectedly discovered in mesencephalic dopaminergic neurons of different mouse strains, as well as in rats and humans affected by Parkinson's disease (PD) and multiple sclerosis (MS) [68–70].

Hb expression is known to decrease in neurons of PD, Alzheimer’s disease (AD), argyrophilic grain disease (AGD) and dementia with Lewy bodies (DLB) brains [44] as well as in the CNS of scrapie-infected mice [13, 14]. Also, it has been shown that Hb binds to Aβ enhancing its aggregation and co-localizes in amyloid plaques in AD brains [71]. If we consider a possible similar interaction with β-rich PrP^Sc^ isoforms in prion diseases, we can hypothesize that in our animal model down-regulated Hb fails to promote aggregation of the prion protein, thus leading to a higher presence of toxic species like oligomers [72]. Moreover, in PD it has been hypothesized that Hb may act as oxygen storage molecule in oligodendrocytes [68]. Oxygen would be later released to neighboring neurons in hypoxia conditions to maintain the aerobic metabolism [68, 69]. When down-regulated, Hb would not be available for this function and cells would be damaged by the defective oxygen homeostasis. Our results indicated a strong down-regulation (about 70-80% lower expression than normal) of both *HBB* and *HBA2* in symptomatic advanced-stage BSE-infected macaques. The data were analyzed with a very stringent procedure after excluding any major effect of potential blood contamination, thus confirming the robustness of the results.

Taken together, all these data indicate a possible general role for hemoglobin in neurodegenerative disorders, possibly related to an alteration of O_2_ homeostasis and oxidative metabolism [68]. One point that needs further investigation is whether this alteration (down-regulation) occurs as an early/late consequence of the disease, or may act as a susceptibility factor that influences the onset of the pathology. Furthermore, future studies may investigate the localization of the observed down-regulation in terms of cell population: it could involve neurons as well as astrocytes or microglia.

Another crucial molecule, APOC1, was significantly up-regulated in BSE-infected brains samples compared to controls. Apolipoprotein C-I, whose gene *APOC1* is part of the APOE/C-I/C-IV/C-II gene cluster, (apoC-I) is a small 6.6 kDa component of lipoproteins (mainly HDL) that is known to inhibit receptor-mediated lipoprotein clearance, especially particles containing apoE [73]. Increasing evidence indicates a role for this gene in neurodegenerative disorders, especially in AD and MS [74–76]. A disruption in lipid metabolism and signaling is one of the early alterations apparent in many neurodegenerative diseases, including prion diseases [77, 78]; indeed, cholesterol metabolites are investigated by a number of studies aimed at the identification of early biomarkers for neurodegenerative disorders [79–81]. Several genes involved in cholesterol metabolism and lipid biosynthesis have been found to be up-regulated in preclinical scrapie-infected mice [64]. Since APOC1 is able to activate cholesterol esterification via lecithin-cholesterol acyltransferase [75], its up-regulation could lead to an increase in cholesterol biosynthesis, consistent with the concomitant presence of prion disease. In fact, *in vitro* studies have shown that depletion of cellular cholesterol reduces the conversion of PrP^C^ to PrP^Sc^[82] and evidence exists also in AD, where altered cholesterol metabolism has been found [83]. Hypercholesterolemia has also been shown to influence amyloid precursor protein processing [84]. One explanation for altered cholesterol homeostasis affecting prion disease development could lie in the fact that PrP is localized in cholesterol-rich lipid rafts [85].

*SERPINA3*, a serpin peptidase inhibitor involved in acute phase response pathways, is another gene that we found highly regulated in our animal model. It is extensively reported to be regulated in other neurodegenerative disease models and in particular it is well known to interact with APP to promote amyloid plaque formation —a hallmark of AD [86]. Indeed, increased levels of SERPINA3 have been found in the brain and peripheral blood of AD patients [87], mainly due to persistent and almost chronic inflammation [88]. In prion disease studies, SERPINA3 was found increased in brains of scrapie-infected mice [77], in mice infected with RML prior to clinical onset [89] as well as in urine and cerebrospinal fluid of CJD patients [90]. Being an acute phase protein, its up-regulation is explained by the onset of an inflammatory condition, particularly as a response of the innate immune system [91]. Interestingly, two β-sheets of SERPINA3 exhibit a polymorphism mimicking changes in the serpin structure that normally occur during the formation of its stable complex with the target proteinase. In this conformation, SERPINA3 can bind Aβ, thus imposing a β-strand conformation that upon dissociation leads to a faster formation of fibrils [86]. Therefore, an intriguing hypothesis may be envisioned in which PrP conversion into β-sheet conformation can be assisted by SERPINA3, which would accelerate the formation of toxic species like PrP oligomers.

Transthyretin, a protein in the same pathway of acute phase response as SERPINA3, was found to be up-regulated at the transcription level in our BSE-infected macaques according to the SYBR® Green assay. Even though we were not able to confirm the statistical significance using the TaqMan® assay, this gene seems to be of interest. Indeed, TTR, carrier of the thyroid hormone thyroxine (T_4_) in serum and CSF, is associated with systemic amyloidosis in humans [92], but also with an anti-amyloidogenic effect preventing Aβ deposition in neuronal cell cultures [93]. Moreover, increased mRNA and protein levels have been shown in neurons from the AD mouse model ‘APP23’ and in human AD brain with a neuroprotective role [94, 95]. Even in prion models *TTR* levels have been found strongly increased in the cortex of scrapie-infected mice [15]. Our study now provides indication that up-regulation of *TTR* may also be found in BSE-infected macaques, further reinforcing the hypothesis of a common mechanism in AD and TSEs. Taken together, these data may suggest innate immune system activation and inflammatory response in these diseases [96], leading to a sustained up-regulation of both *SERPINA3* and *TTR* genes simultaneously: *SERPINA3* as inflammation effect, *TTR* as attempt to neutralize the infectious agent preventing its deposition. However, analysis of the microarray data did not reveal relevant deregulation of other genes typically involved in neuroinflammation and/or immune response, such as cytokines and other mediators. Even though some authors have reported alteration of these pathways [97], in our array IL6, TNFα, GFAP and CD68 showed a fold change < |2|, suggesting that inflammatory responses may not be particularly severe in this model.

One last point that remains to be addressed is the expression of the prion protein gene itself (*PRNP* in humans) upon infection. Because of shortage of cDNA, we were not able to validate its levels in our samples. Nevertheless, our microarray data did not identify any changes between control and infected samples, at least at the mRNA level. This is in agreement with findings reported for BSE-infected cattle [3], but differs from the situation in sporadic CJD patients who show reduced mRNA expression [97]. Whether this disagreement is related to the host or the infectious agent needs to be explored.

## Conclusions

To our knowledge, this is the first genome-wide expression study in the *gyrus frontalis superior* region of cynomolgus macaques inoculated with BSE. Using microarray and RT-qPCR technologies we identified a gene signature able to distinguish infected macaques from control animals. These results could be extremely helpful in understanding the progression of the disease, allowing for the identification of some key players which, if not being the cause of the onset, could be some of the target genes affected by the disease. Therefore, after deeper investigations to validate these targets at the protein level and confirm their specificity for prion diseases, they may be exploited as potential biomarkers to set up pre-clinical diagnostic tests.

In particular, our findings support the hypothesis of a potential shared mechanism underlying the onset and the development of all neurodegenerative disorders, as the majority of our DEGs are known to be involved in other diseases such as AD or PD. This is in concordance with very recent data supporting the idea of a unifying role of prions in these diseases in general and maybe a prion-like behavior for most neurodegenerative disorders [98]. Furthermore, some of the DEG transcripts we found are present also in blood (hemoglobin, transthyretin, serpin peptidase inhibitor) and among them hemoglobin exhibited decreased expression throughout the entire course of the infection, including preclinical time points, in mouse models. Therefore, there is the intriguing possibility to employ these "readily available" biomarkers for diagnostic purposes, especially if additional studies will confirm the expression level of the proteins encoded by these DEGs in brain and/or blood tissue.

In general, our results suggest that, in order to identify potential biomarkers and drug targets for prion diseases and other neurodegenerative disorders, a combination of various pathways has to be targeted, including oxygen homeostasis, lipid metabolism and inflammation response.

In summary, large-scale transcriptome analyses of human TSEs are rare [97, 99] and primate models are a valid approach to better understand the mechanisms of these fatal diseases. Even with all the limitations discussed above, our BSE-infected macaques are, to our knowledge, the closest available model for human vCJD and these results, obtained with an unbiased methodology as the gene expression microarray technology, are contributing to shed some light on the molecular basis of TSEs as well as neurodegeneration as a whole.

## Methods

### Ethics statement

Ethics approval for the study was issued by the Lower Saxony Ministry for consumer protection and food safety (509.42502/08/07.98). Animal experimentation was performed in accordance with section 8 of the German Animal Protection Law in compliance with EC Directive 86/609.

### Samples

Samples were derived from six BSE-infected macaques, *Macaca fascicularis* (A1 to A6) that were intracranially inoculated with a single dose of 50 mg brain homogenate (10% wt/vol) [33, 37]. One cynomolgus macaque (B6) was orally inoculated with the same material; inoculation was performed *per os*, as single dose.

Brain material from five age- and sex-matched non-infected cynomolgus macaques (CovA, CovB, CovC, CovD1, CovD2) was obtained from Covance Laboratory Münster GmbH and processed in an equivalent manner.

### Tissue and RNA extraction

At autopsy of seven BSE-infected cynomolgus macaques at advanced stage of disease and five non-infected control animals, one hemisphere of the brain was sliced dorso-ventrally and snap-frozen on dry-ice plates. The *gyrus frontalis superior* region was macroscopically identified on the frozen tissue and removed via a biopsy stamp. Total RNA (RNA > 200 bases) was isolated by manually homogenizing the material with micro pestles (Kisker Biotech GmbH) in TRIzol (Invitrogen). RNA isolation was performed according to the supplier’s instructions. Following RNA isolation, a DNase I digestion was performed using 1 unit of enzyme per μg RNA (Fermentas) for 30 min at 37°C, and heat inactivated for 5 min at 95°C followed by precipitation with Sodium Acetate/Ethanol. RNA was checked for quantity and purity on a Spectrophotometer 2000 (PEQLAB) and for integrity of the 18S and 28S ribosomal bands by capillary electrophoresis using the 2100 Bioanalyzer (Agilent Technologies).

### Immunoblot analysis

PK-treated (50 μg/mL for 1 hour at 37°C) and untreated brain homogenates corresponding to 0.7 mg or 0.3 mg brain tissue, respectively, were separated on 12% Bis/Tris Acrylamide gels (NuPAGE, Invitrogen) and transferred to nitrocellulose membranes (Protran, Schleicher & Schüll, Germany). Detection of macaque PrP^Sc^ was performed using the monoclonal anti-PrP antibody 11C6 and a Peroxidase conjugated anti-mouse IgG-antibody (Sigma-Aldrich, Germany). Signal was visualized using a chemiluminescent substrate (Super Signal West Pico, Pierce) and high sensitivity films (Amersham). Densitometric analysis of PrP^Sc^ was performed using the Image J program 1.37v.

### Microarray analysis using the GeneChip® Rhesus Macaque genome array

Samples were labeled using the GeneChip® 3’IVT Express Kit (Affymetrix®). Reverse transcription of RNA was performed using 500 ng of total RNA to synthesize first-strand cDNA. This cDNA was then converted into a double-stranded DNA template for transcription. *In vitro* transcription included a linear RNA amplification (aRNA) and the incorporation of a biotin-conjugated nucleotide. The aRNA was then purified to remove unincorporated NTPs, salts, enzymes, and inorganic phosphate. The labeled aRNA of each animal was fragmented (50–100 bp) and hybridized to a GeneChip® Rhesus Macaque Genome Array (Cat N° 900656; Affymetrix®). The degree of fragmentation and the length distribution of the aRNA were checked by capillary electrophoresis using the Agilent 2100 Bioanalyzer (Agilent Technologies).

The hybridization was performed for 16 h at 1 × *g* and 45°C in the GeneChip® Hybridization Oven 640 (Affymetrix®). Washing and staining of the arrays were performed on the Gene Chip® Fluidics Station 450 (Affymetrix®) according to the manufacturer's recommendations. The antibody signal amplification and washing and staining protocol were used to stain the arrays with streptavidin R-phycoerythrin (SAPE; Invitrogen). To amplify staining, SAPE solution was added twice with a biotinylated anti-streptavidin antibody (Vector Laboratories, Burlingame, CA, USA) staining step in-between. Arrays were scanned using the GeneChip® Scanner 3000 7G (Affymetrix®).

### Microarray data analysis

Intensity data from the CEL. files were imported to the Partek® software including a quality control based on internal controls. All chips passed the quality control and were analyzed using the Limma package [100] of Bioconductor [101, 102] and the Partek® software. The microarray data discussed in this paper were generated conforming to the MIAME guidelines and are deposited in the NCBI’s Gene Expression Omnibus (GEO) database [103]. They are accessible through GEO series accession number GSE52436 (see section: Availability of supporting data).

The microarray data analysis consisted of the following steps: 1. quantile method normalization, 2. global clustering and PCA-analysis, 3. fitting the data to a linear model, 4. detection of differential gene expression and 5. over-representation analysis of differentially expressed genes. Quantile-normalization was applied to the log2-transformed intensity values as a method for between-array normalization to ensure that the intensities had similar distributions across arrays.

For cluster analysis, we used a hierarchical approach with the average linkage-method. Distances were measured as 1 - Pearson's Correlation Coefficient. The PCA was performed using the princomp-function in the Partek® software. To estimate the average group values for each gene and assess differential gene expression, a simple linear model was fitted to the data, and group-value averages and standard deviations for each gene were obtained. To find genes with significant expression changes between groups, empirical Bayes statistics were applied to the data by moderating the standard errors of the estimated values [100].

P-values were obtained from the moderated t-statistic and corrected for multiple testing with the Benjamini–Hochberg method [104]. The p-value adjustment guarantees a smaller number of false positive findings by controlling the false discovery rate (FDR). For each gene, the null hypothesis, that there is no differential expression between degradation levels, was rejected when its FDR was lower than 0.05. Because no candidates appeared using FDR 0.05, we made the selection using another p-value (unadjusted p-value ≤ 0.005) and a fold change ≥ |2|.

### Reverse transcription and RT-qPCR

Validation by quantitative reverse transcription real-time PCR (RT-qPCR) was performed using gene-specific primer pairs. cDNA synthesis was accomplished using 100 ng RNA, 10 ng random hexamer primer, 2 mM dNTPs, 0.5 U RNase inhibitor and 5 U reverse transcriptase (Bioline) in 1× reaction buffer. For each sample a negative control was carried along by omission of the reverse transcriptase (−RT control).

The cDNA was diluted 1:10 prior to RT-qPCR. Ten ng RNA equivalent was added to the reaction mix including 2× iQ™ SYBR® Green Supermix (Bio-Rad Laboratories, Inc.), 400 nM of the corresponding forward and reverse primer (Sigma), and quantified in technical triplicates on an iQ5 Multicolor Real-Time PCR Detection System (Bio-Rad Laboratories, Inc.). All primers used for RT-qPCR are listed in Table 3.

After initial denaturation for 3 min at 95°C, 45 cycles were performed at 95°C for 15 sec and 58°C for 1 min. Differential gene expression of candidates was normalized to *GAPDH* and *ACTB* expression. –RT controls were included in the plates for each primer pair and sample. The relative expression ratio was calculated using the ΔΔC_T_ method [105, 106]. Significance was calculated with the unpaired student *t*-test (p < 0.05). Melting curve analysis and gel electrophoresis of amplification products were performed for each primer pair to verify that artificial products or primer dimers were not responsible for the signals obtained. Some results were further confirmed using TaqMan® MGB probes and iQ™ Multiplex Powermix (Bio-Rad Laboratories, Inc.). The primer sequences, the reaction setup and the cycling conditions were the same as described above.

The probe sequences used for the detection of specific targets were:

- GAPDH: 5’-FAM CTGGCCAAGGTCATCCATGA-3’;
- ACTB: 5’-FAM-ACAAGATGAGATTGGCATGGC-3’;
- HBB: 5’-FAM-AAGTGCTTGGTGCCTTTAGTGATGG-3’;
- HBA2: 5’-FAM-TGGCGAGTATGGTGCGGAGG-3’;
- SERPINA3: 5’-FAM-TTCCTGGCCCCTGTGATCCC-3’;
- TTR: 5’-FAM-ATCGTTGGCTGTGAATACCACCTCTG-3’;
- APOC1: 5’-FAM-TGGAGGACAAGGCTTGGGAAGTG-3’.

### Availability of supporting data

The microarray data set supporting the results of this article is available in the Gene Expression Omnibus (GEO) repository, [http://www.ncbi.nlm.nih.gov/geo/query/acc.cgi?token=wnmjowqqhrcpzod&acc=GSE52436].

The DEGs were analyzed for their functions, pathways and networks using Ingenuity Pathways Analysis-IPA® [http://www.ingenuity.com/products/ipa/try-ipa-for-free].

## Electronic supplementary material

Additional file 1: **List of 86 differentially expressed probe sets with p values ≤ 0.005 and FC ≥ │2│.** Probe ID, Gene Symbol, Gene Name and RefSeq Transcript IDs annotation as of release 29 of the Affymetrix® Rhesus Annotation library (01/July/09). P-values and fold changes are reported for all 86 probe sets. (XLSX 18 KB)

Additional file 2: **List of 97 differentially expressed probe sets selected as RT-qPCR candidates.** Probe IDs and Previous Gene Symbol annotation as of release 29 of the Affymetrix® Rhesus Annotation library (01/July/09). Current Gene Symbol annotation as of the latest Affymetrix® Rhesus Annotation library (release 32 - 09/June/11). Gene Name and RefSeq Transcript IDs as of Ensembl release 72 (June 2013). Annotation using alignment with the human genome has been performed (as stated in the gene name column) for the most highly regulated probe sets with unknown macaque annotation. P-values and fold changes are reported for all genes. (XLSX 57 KB)

Additional file 3: **Evaluation of reference gene expression stability across non-infected and BSE-infected samples.** For each sample, average values of absolute C_Ts_ (+/−SD) of triplicate wells for *GAPDH* (grey) and *ACTB* (white) are shown. (PDF 47 KB)

Additional file 4: Δ**C**_**T**_ **values for all genes showing variability among BSE-infected samples.** ΔC_T_ values (+/−SD) normalized against *GAPDH*. Very similar results were obtained with normalization against *ACTB* (data not shown). (PDF 58 KB)

Additional file 5: Δ**C**_**T**_ **values for all genes showing variability among non-infected samples.** ΔC_T_ values (+/−SD) normalized against *GAPDH*. Very similar results were obtained with normalization against *ACTB* (data not shown). (PDF 59 KB)

Additional file 6: ΔΔ**C**_**T**_ **values of selected genes in the infected samples.** ΔΔC_T_ values (+/−SD) for *HBB*, NR4A2, *NCAM1*, *USP16* and *AKR1C1* normalized against *GAPDH* in the orally-infected animal B6 (white) compared to intracranially infected samples A1-A6 (grey). Only 5 genes were analyzed for animal B6 due to shortage of cDNA. (PDF 27 KB)

Additional file 7: **Cluster analysis.** Cluster analysis was performed using a hierarchical approach with the average linkage-method for all animals (panel A) or excluding the orally infected one, B6 (panel B). (PDF 68 KB)

Additional file 8: **SYBR® Green-based RT-qPCR validation of microarray results.** Relative expression levels of 11 genes in BSE-infected cynomolgus macaques normalized against *ACTB* as reference gene. (PDF 47 KB)

Additional file 9: **Comparison between SYBR® Green -based and TaqMan® probe-based results for** ***TTR.*** Average values of absolute C_Ts_ (+/− SD) of triplicate wells for *TTR* obtained with SYBR® Green (grey) and TaqMan® probe (white) detection methods in BSE-infected samples are shown. (PDF 29 KB)

Additional file 10: **RT-qPCR analysis of blood specific marker** ***RHAG.*** C_T_ values for the erythrocyte marker *RHAG* were monitored across BSE-infected (solid fill) and non-infected (dotted fill) samples. Human blood cDNA was used as positive control (gradient fill). Note that for almost all the samples C_T_ values were ≥ 35 therefore indicating a very low expression level. Primer sequence (3’-5’): RHAG: F = AGGCAAGCTCAACATGGTTC, R = GGGTGAATTGCCATATCCGC. (PDF 56 KB)

Additional file 11: **RT-qPCR analysis of blood specific marker** ***ALAS2.*** C_T_ values for the erythrocyte marker *ALAS2* were monitored across BSE-infected (solid fill) and non-infected (dotted fill) samples. Human blood cDNA was used as positive control (gradient fill). Note that for almost all the samples C_T_ values were ≥ 35 therefore indicating a very low expression level. Primer sequence (3’-5’): ALAS2: F = TCCCTTCATGCTGTCGGAAC, R = GAGCTAGGCAGATCTGTTTTGAA. (PDF 56 KB)

## Abbreviations

RT-qPCR: Reverse transcriptase quantitative polymerase chain reaction
HBB: Hemoglobin, beta
HBA2: Hemoglobin, alpha 2
TTR: Transthyretin
APOC1: Apolipoprotein C-I
SERPINA3: serpin peptidase inhibitor, clade A (alpha-1 antiproteinase, antitrypsin), member 3
TSE: Transmissible spongiform encephalopathy
CJD: Creutzfeldt-Jakob disease
GSS: Gerstmann-Sträussler-Scheinker syndrome
FFI: Fatal familial insomnia
BSE: Bovine spongiform encephalopathy
CWD: Chronic wasting disease
FSE: Feline spongiform encephalopathy
PrP^C^: Cellular prion protein
PrP^Sc^: Scrapie prion protein
PRNP: Prion protein
ER: Endoplasmic reticulum
DEG: Differentially expressed gene
RIN: RNA integrity number
FDR: False discovery rate
IPA: Ingenuity pathways analysis
GAPDH: Glyceraldehyde-3-phosphate dehydrogenase
ACTB: Actin, beta
AKR1C1: Aldo-keto reductase family 1, member C1
NCAM1: Neural cell adhesion molecule 1
USP16: Ubiquitin specific peptidase 16
NR4A2: Nuclear receptor subfamily 4, group A, member 2
ALAS2: Aminolevulinate, delta-, synthase 2
RHAG: Rh-associated glycoprotein
FC: Fold change
Hb: Hemoglobin
SN: Substantia nigra
PD: Parkinson's disease
MS: Multiple sclerosis
AD: Alzheimer’s disease
AGD: Argyrophilic grain disease
DLB: Dementia with Lewy bodies
Hpt: Haptoglobin
CNS: Central nervous system
HDAC: Histone deacetylase
APP: Amyloid beta precursor protein
PS1: Presenilin 1
RML: Rocky mountain laboratory
Aβ: Amyloid beta
HDL: High density lipoprotein
LDL: Low density lipoprotein
APOE: Apolipoprotein E
LOAD: Late onset Alzheimer’s disease
IRF3: Interferon regulatory factor 3
vCJD: Variant Creutzfeldt-Jakob disease
DNase I: Deoxyribonuclease I
dNTP: 2'-deoxynucleoside 5'-triphosphate
MGB: Minor groove binder
PCA: Principal component analysis
PK: Proteinase K.
