# Supplementary figures and images for: Gene expression profiling of brains from bovine spongiform encephalopathy (BSE)-infected cynomolgus macaques
Source: BMC Genomics. 2014 Jun 5;15:434. doi: 10.1186/1471-2164-15-434 (PMC4061447; doi:10.1186/1471-2164-15-434)

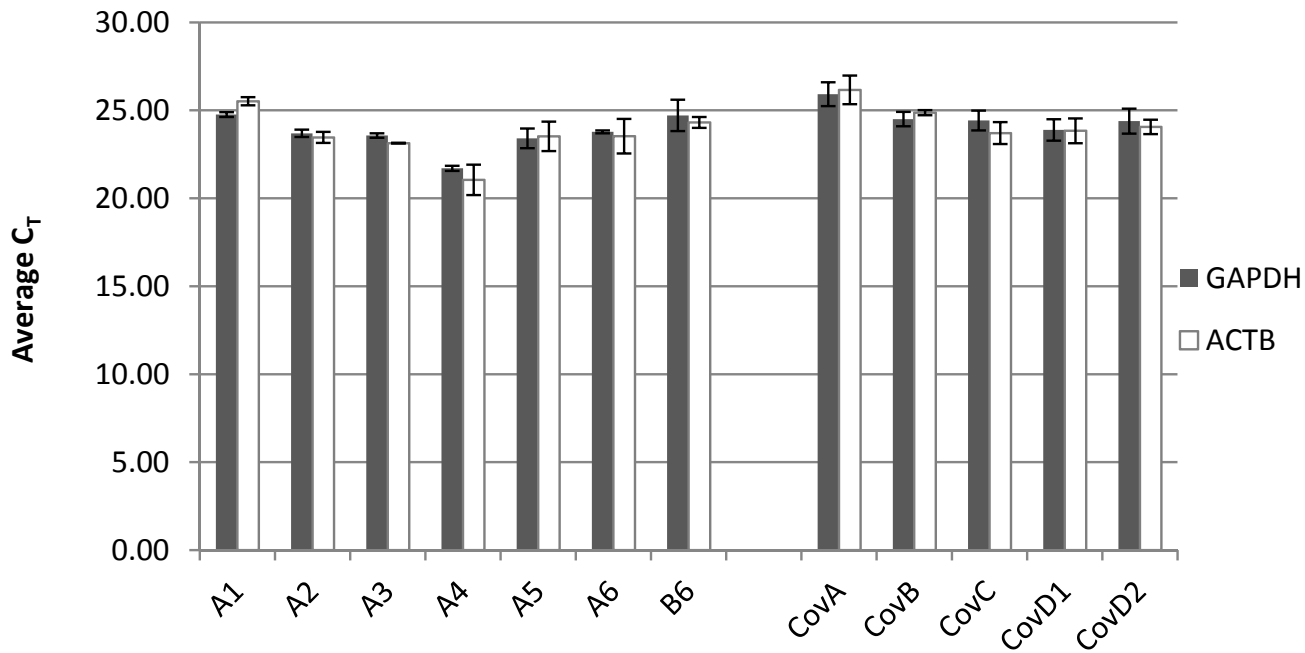

Supplement: Supplementary file 3 — Additional file 3: Evaluation of reference gene expression stability across non-infected and BSE-infected samples. For each sample, average values of absolute CTs (+/−SD) of triplicate wells for GAPDH (grey) and ACTB (white) are shown. (PDF 47 KB) [file 12864_2014_6117_MOESM3_ESM.pdf]

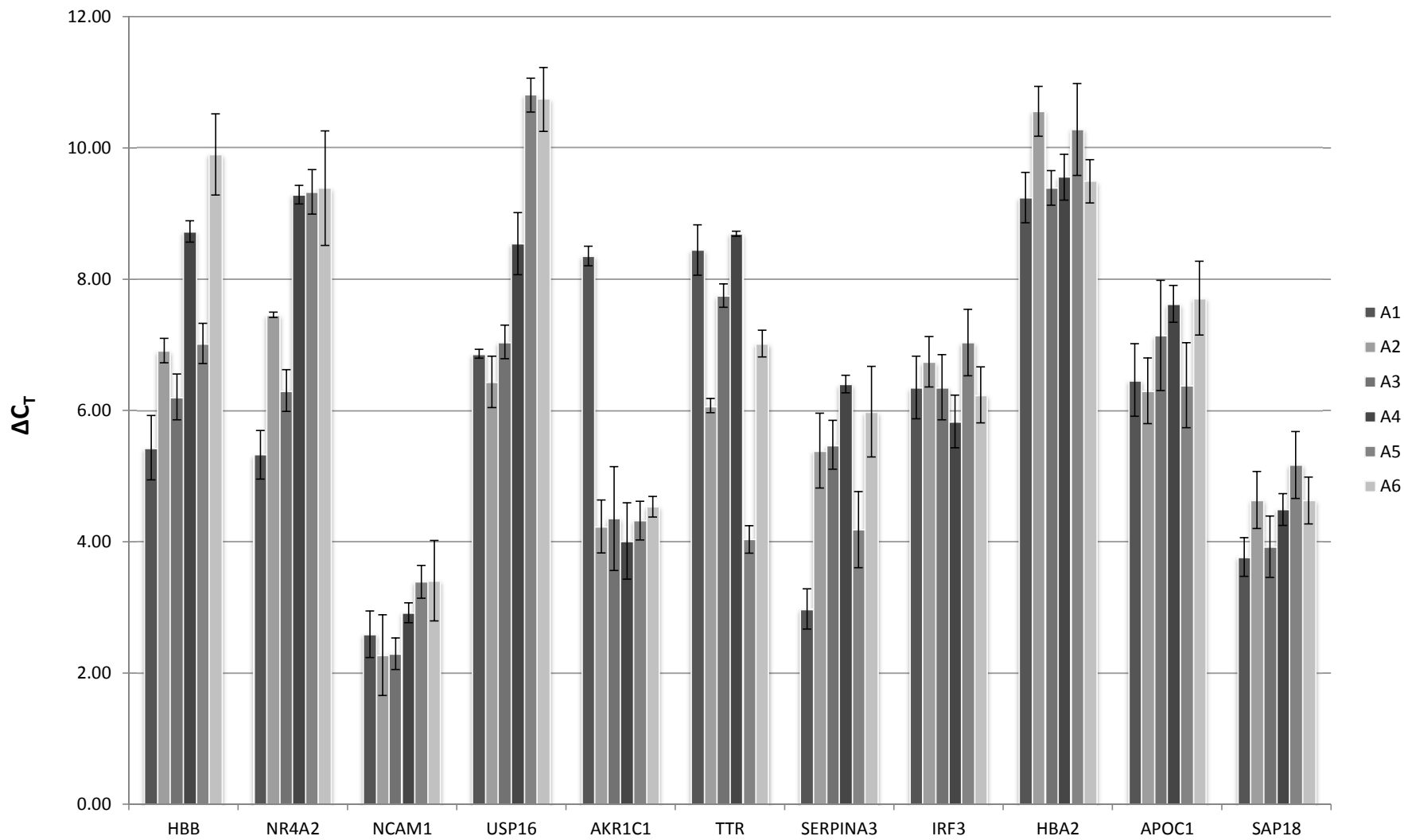

Supplement: Supplementary file 4 — Additional file 4: ΔC T values for all genes showing variability among BSE-infected samples. ΔCT values (+/−SD) normalized against GAPDH. Very similar results were obtained with normalization against ACTB (data not shown). (PDF 58 KB) [file 12864_2014_6117_MOESM4_ESM.pdf]

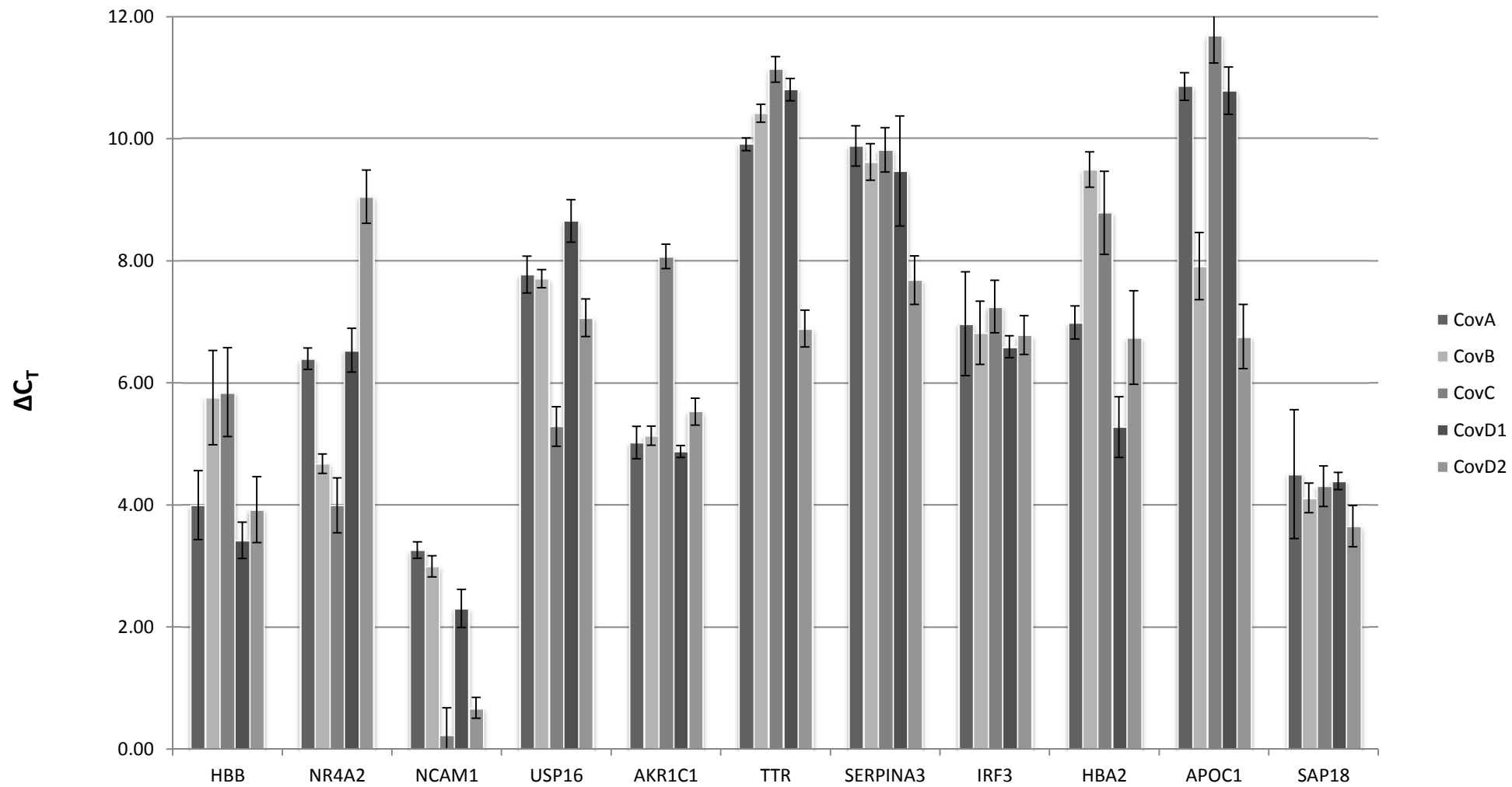

Supplement: Supplementary file 5 — Additional file 5: ΔC T values for all genes showing variability among non-infected samples. ΔCT values (+/−SD) normalized against GAPDH. Very similar results were obtained with normalization against ACTB (data not shown). (PDF 59 KB) [file 12864_2014_6117_MOESM5_ESM.pdf]

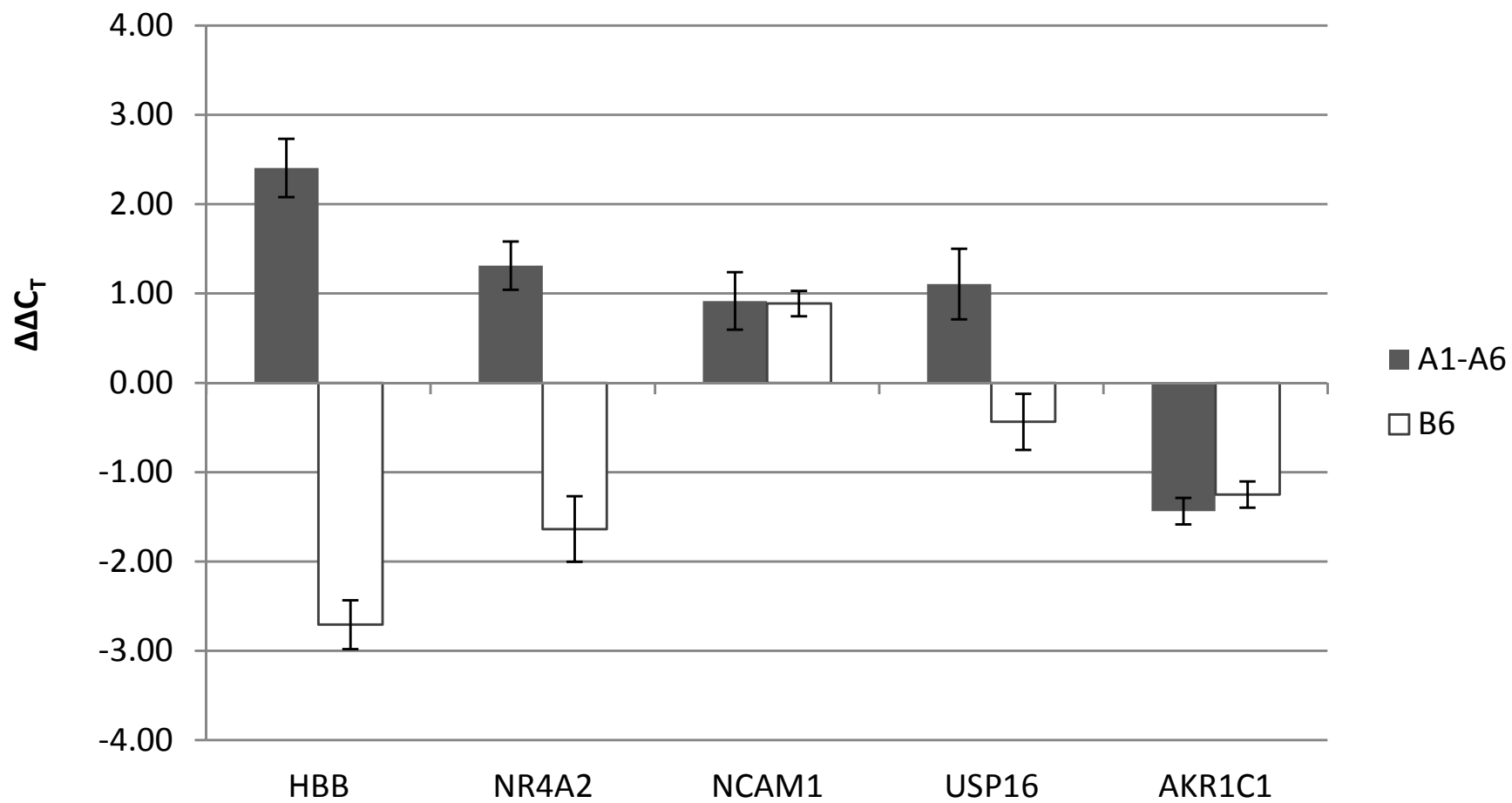

Supplement: Supplementary file 6 — Additional file 6: ΔΔC T values of selected genes in the infected samples. ΔΔCT values (+/−SD) for HBB, NR4A2, NCAM1, USP16 and AKR1C1 normalized against GAPDH in the orally-infected animal B6 (white) compared to intracranially infected samples A1-A6 (grey). Only 5 genes were analyzed for animal B6 due to shortage of cDNA. (PDF 27 KB) [file 12864_2014_6117_MOESM6_ESM.pdf]

**A**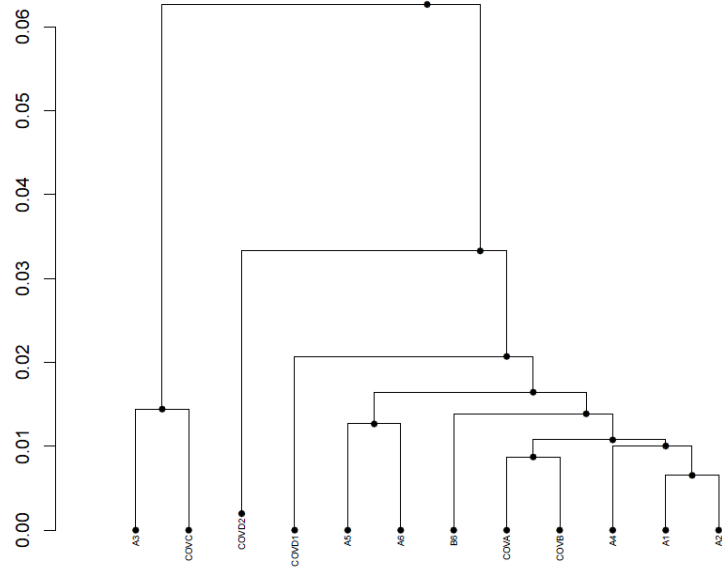**B**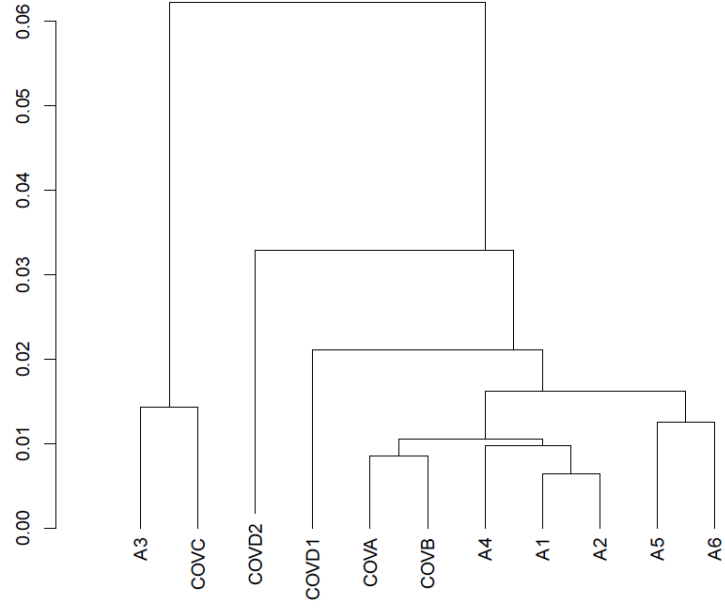

Supplement: Supplementary file 7 — Additional file 7: Cluster analysis. Cluster analysis was performed using a hierarchical approach with the average linkage-method for all animals (panel A) or excluding the orally infected one, B6 (panel B). (PDF 68 KB) [file 12864_2014_6117_MOESM7_ESM.pdf]

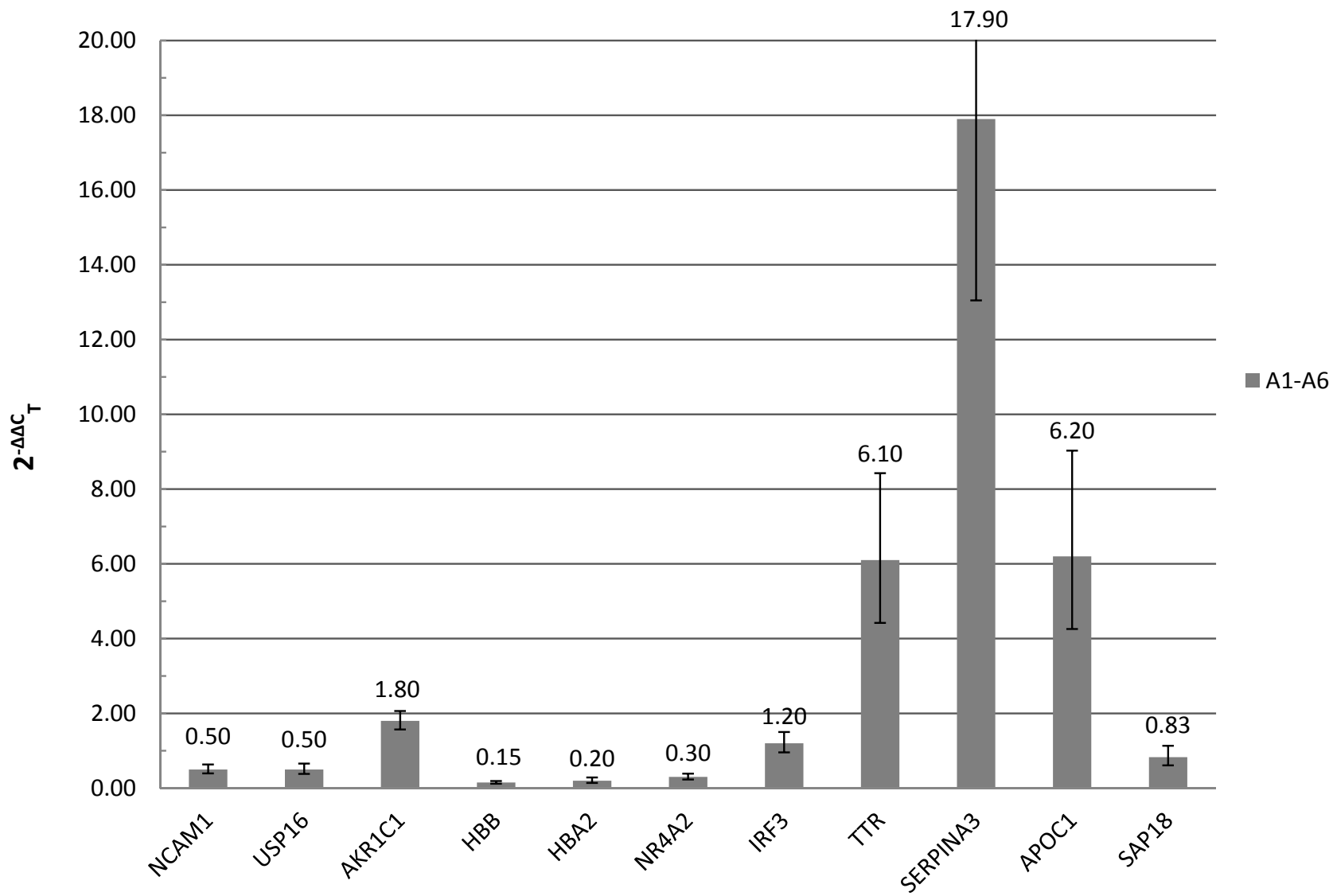

Supplement: Supplementary file 8 — Additional file 8: SYBR® Green-based RT-qPCR validation of microarray results. Relative expression levels of 11 genes in BSE-infected cynomolgus macaques normalized against ACTB as reference gene. (PDF 47 KB) [file 12864_2014_6117_MOESM8_ESM.pdf]

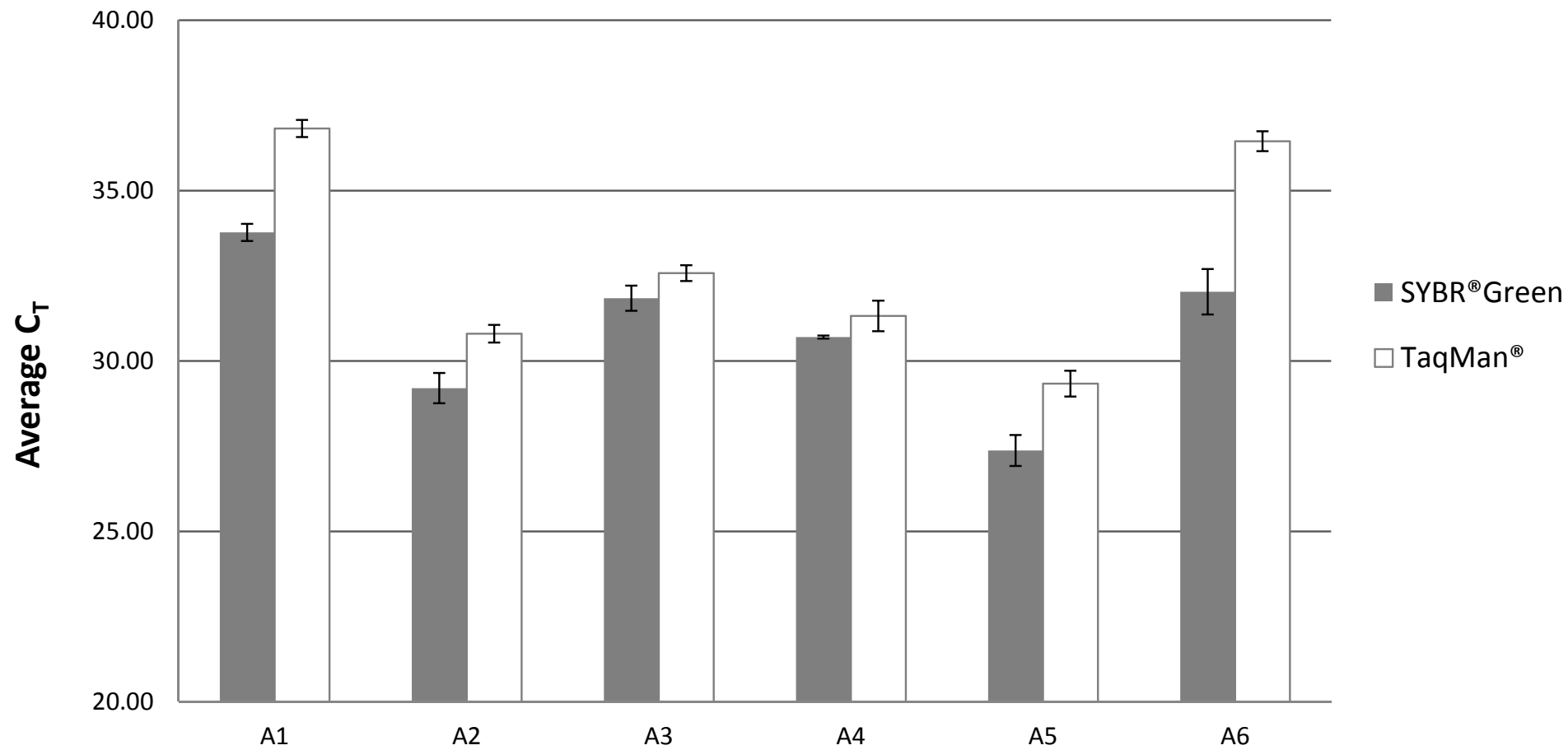

Supplement: Supplementary file 9 — Additional file 9: Comparison between SYBR® Green -based and TaqMan® probe-based results for TTR. Average values of absolute CTs (+/− SD) of triplicate wells for TTR obtained with SYBR® Green (grey) and TaqMan® probe (white) detection methods in BSE-infected samples are shown. (PDF 29 KB) [file 12864_2014_6117_MOESM9_ESM.pdf]

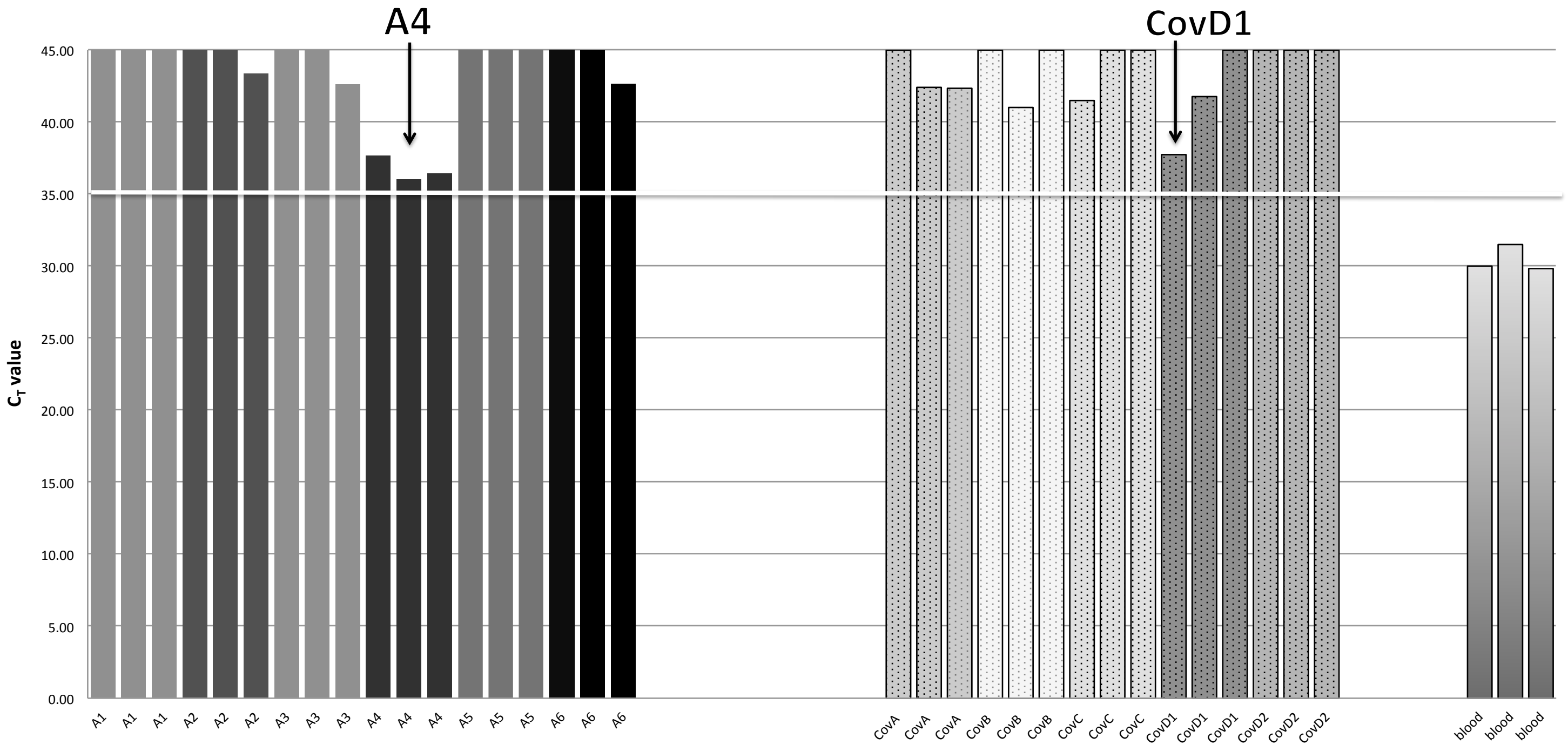

Supplement: Supplementary file 10 — Additional file 10: RT-qPCR analysis of blood specific marker RHAG. CT values for the erythrocyte marker RHAG were monitored across BSE-infected (solid fill) and non-infected (dotted fill) samples. Human blood cDNA was used as positive control (gradient fill). Note that for almost all the samples CT values were ≥ 35 therefore indicating a very low expression level. Primer sequence (3’-5’): RHAG: F = AGGCAAGCTCAACATGGTTC, R = GGGTGAATTGCCATATCCGC. (PDF 56 KB) [file 12864_2014_6117_MOESM10_ESM.pdf]

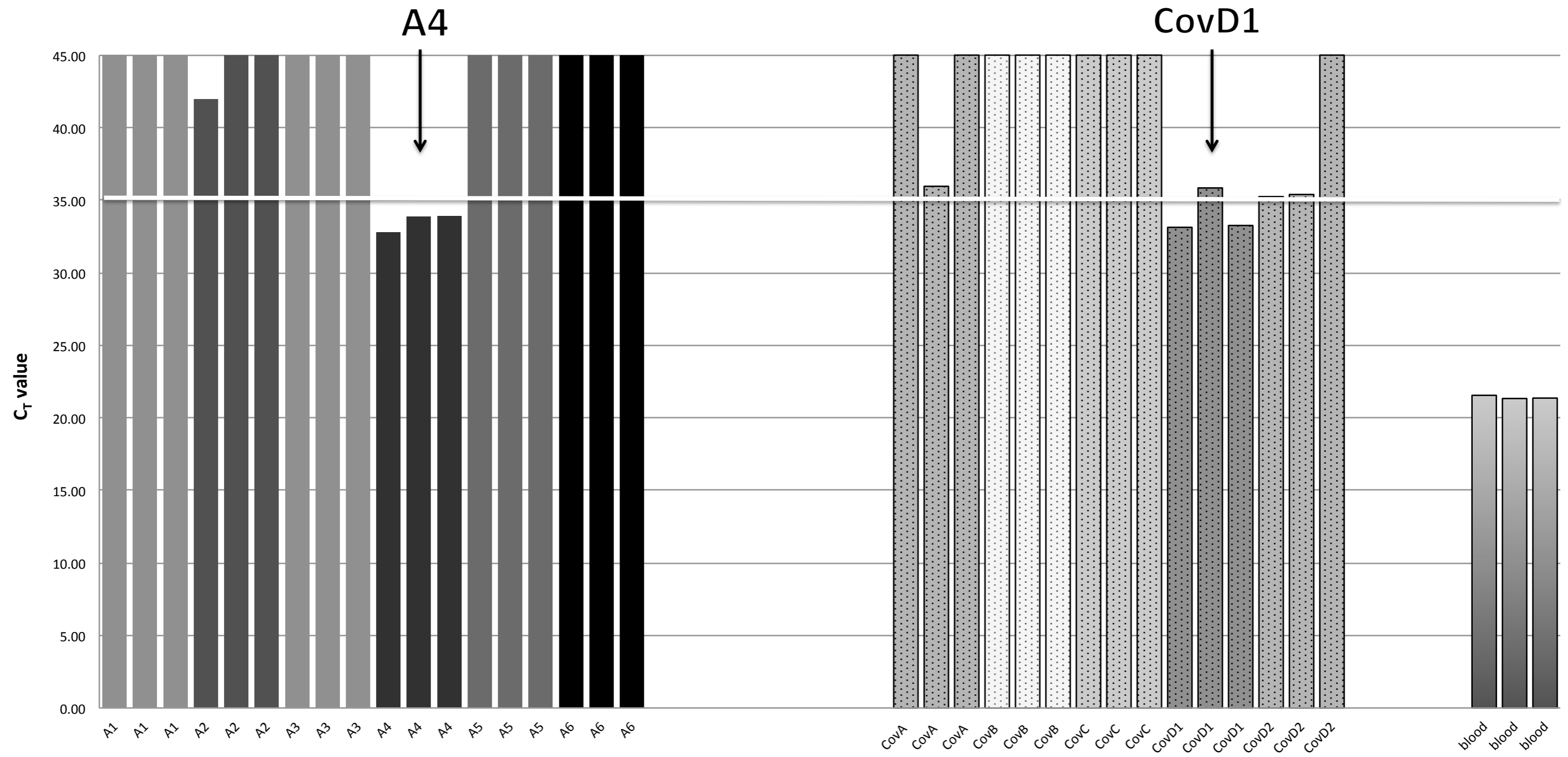

Supplement: Supplementary file 11 — Additional file 11: RT-qPCR analysis of blood specific marker ALAS2. CT values for the erythrocyte marker ALAS2 were monitored across BSE-infected (solid fill) and non-infected (dotted fill) samples. Human blood cDNA was used as positive control (gradient fill). Note that for almost all the samples CT values were ≥ 35 therefore indicating a very low expression level. Primer sequence (3’-5’): ALAS2: F = TCCCTTCATGCTGTCGGAAC, R = GAGCTAGGCAGATCTGTTTTGAA. (PDF 56 KB) [file 12864_2014_6117_MOESM11_ESM.pdf]
